# Chronic Progressive Lymphedema in Belgian Draft Horses: Understanding and Managing a Challenging Disease

**DOI:** 10.3390/vetsci10050347

**Published:** 2023-05-12

**Authors:** Marieke Brys, Edwin Claerebout, Koen Chiers

**Affiliations:** 1 Laboratory of Veterinary Pathology, Department of Pathobiology, Pharmacology and Zoological Medicine, Faculty of Veterinary Medicine, Ghent University, Salisburylaan 133, 9820 Merelbeke, Belgium; koen.chiers@ugent.be; 2 Laboratory of Parasitology, Faculty of Veterinary Medicine, Ghent University, Salisburylaan 133, 9820 Merelbeke, Belgium; edwin.claerebout@ugent.be

**Keywords:** Belgian draft horse, clinical signs, skinfolds, fibrosis, pathogenesis, lymphedema, elastin, hyperkeratosis, treatment

## Abstract

**Simple Summary:**

Chronic progressive lymphedema (CPL) is a condition with a significant impact on the health and welfare of the current draft horse population. Clinical signs are severe and primarily involve the progressive swelling of the distal portions of the legs, which is accompanied by scaling, marked dermal fibrosis, and the development of skinfolds and nodules, which are often complicated by secondary infections. Current treatment and management options aim only at slowing down the disease’s progression. Despite the severity of this condition, many uncertainties regarding its etiology and pathogenesis still persist to date. Understanding and recognizing the clinical signs, as well as managing CPL, therefore, remain major challenges in current veterinary practice. This review discusses possible hypotheses for the pathogenesis of CPL in order to gain new insights and provide a clear perspective for future research. We also cover advances in CPL diagnosis and management, along with an illustrated overview of primary, secondary, and CPL-associated lesions. Furthermore, a renewed scoring system has been developed, which is based on new insights, to assess and grade clinical CPL severity in draft horses by using a uniform and simplified method of evaluation.

**Abstract:**

Chronic progressive lymphedema (CPL) in draft horses is characterized by increased dermal thickness and fibrosis, with the development of skinfolds and nodules, hyperkeratosis, and ulcerations on the distal limbs of affected horses. Secondary bacterial, fungal, or parasitic infections frequently complicate and aggravate the lesions, as well as the progression of this disease. CPL has a particularly high prevalence of up to 85.86% in the Belgian draft horse breed. Due to the disease’s progressive and incurable nature, affected horses are often euthanized prematurely. The treatment options are solely symptomatic, aimed at improving the horse’s quality of life. Despite the severity of this condition, many uncertainties about its etiology and pathogenesis still remain to date. The established scientific research on CPL is rather limited, although there is an urgent need for strategies to tackle this disease. This review summarizes the available knowledge, serving as a guideline for practitioners, and provides perspectives for future research programs.

## 1. Introduction

The Belgian draft horse is a world-renowned breed due to its significant historical importance in agriculture at the beginning of the 20th century. In 2018, it was recognized as a national intangible cultural heritage in Belgium. However, currently, high inbreeding and increasing coancestry rates are putting the future existence of the breed at serious risk. Furthermore, the vulnerable population of Belgian draft horses has been compromised by an incurable disorder; chronic progressive lymphedema (CPL). The first reports of CPL date back to the early 1900s, and despite this, there are still many uncertainties about this condition, and CPL remains incurable at present [1,2]. The supportive treatment for CPL is time-consuming and only aims at improving the quality of the horse’s life. As the clinical signs progressively aggravate, CPL often results in premature euthanasia, markedly decreasing the average life expectancy in the Belgian draft horse [3,4,5]. 

**Figure 1:**
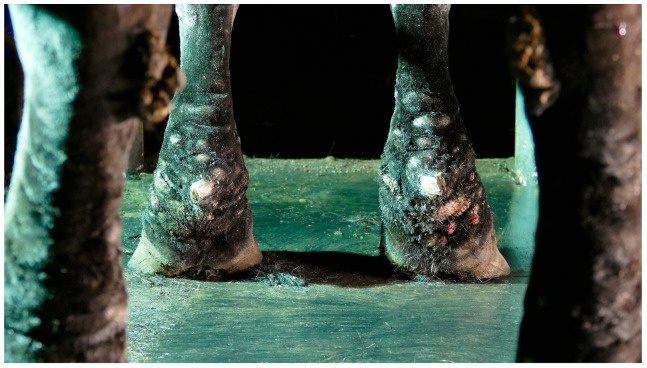
Typical lesions of CPL on the distal legs of a Belgian draft horse. Characterizing skinfolds and nodules may be observed on the distal legs, associated with scaling of the skin (after clipping of the feathering).

CPL is characterized by a progressive swelling of the distal portions of the legs, accompanied by scaling, marked dermal fibrosis, and the development of skinfolds and nodules (Figure 1). Secondary recurrent bacterial and parasitic infections often complicate these lesions. This condition was originally termed ‘Chronic Pastern Dermatitis’ (CPD) or ‘Chronic Proliferative Pastern Dermatitis’ (CPPD). In 2003, the syndrome was redefined as ‘Chronic Progressive Lymphedema (CPL), mainly because of its remarkable clinical and histological similarity to non-filarial chronic lymphedema or elephantiasis nostras verrucosa in humans [6]. Today, both C(P)PD and CPL are commonly used in scientific literature to describe the same clinical presentation. The term CPL can be preferred over CPD, since the associated clinical lesions may not be confined to a dermatological problem but probably involve the lymphatic system as well, given that reduced lymphatic clearance has been demonstrated in the limbs of clinically affected draft horses [7,8]. However, chronic progressive lymphedema might also not be an accurate designation for this disorder, since the etiology and pathogenesis of this condition remain speculative to date. Dysfunction of the lymphatic system, progressive fibrosis of the tissues, and a disruption of the elastin matrix in the skin all appear to be key components within this pathology, although further research is required to establish the exact chronology of these events within the pathogenesis of CPL. Lymphedema may well be a contributing factor rather than a causative one. Nonetheless, in this review, the term CPL will be used since this term is widely used in both literature and practice.

De Keyser et al. established a prevalence of 85.86% in the Belgian draft horse population, demonstrating the significant impact of CPL on the conservation of the breed [9]. CPL is considered a multifactorial disorder with genetic susceptibility [9,10]. A genetic trait is suspected since Belgian draft horses were incorporated as ancestors in draft horse breeds all over the world, and all these breeds now appear to be affected by CPL [11,12]. Moreover, CPL also seems to be more prevalent in certain bloodlines. However, additional selection against a genetic disorder will be challenging without a further limiting genetic variation in the population. 

Not only the Belgian draft horse is considered an affected breed; in fact, the condition has also been frequently observed in other breeds, such as in the Shires and Clydesdales [6,13], Gypsy Cobs and Gypsy Vanners [8,14], Friesians [15], the American Belgian [12], German draft horse breeds (the South German, the Black Forest, the Schleswig, the Saxon-Thuringian, the Rhenish-German, and the Mecklenburg) [16], the Percheron [5,17], the Ardennes, the Breton and Boulonnais [5,18], the Cheval de Trait Auxois (personal communication with owners, 2021), the Trait du Nord, the Trait Mulassier Poitevin, and the Comtois [19], as well as in several crossbreeds from the aforementioned breeds. Although no population-wide studies have been conducted on these susceptible breeds, the disease prevalence is expected to be high [4,6,13,14,20]. The emerging prevalence in multiple breeds requires practical management, and thus, it is of great importance to gain a better understanding of the etiology, pathogenesis, and possible diagnostics of this disease.

## 2. Pathogenesis: Two Diverging Hypotheses

**Figure 2:**
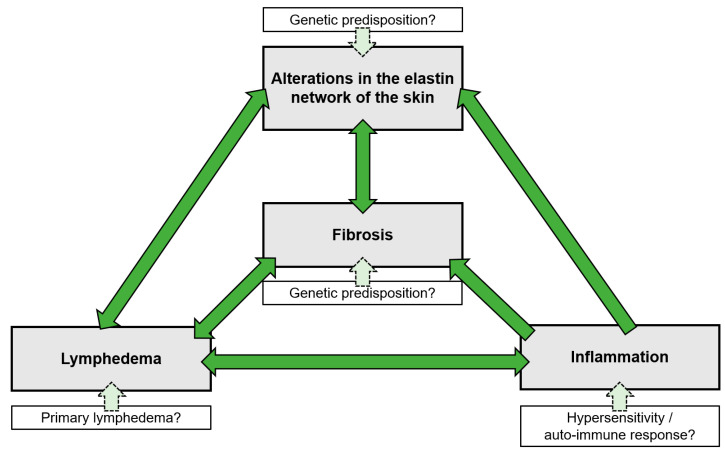
The vicious cycle of CPL. All factors associated with CPL are interconnected and reinforce each other, with the initiating or causative factor in this cycle still remaining speculative. A genetic factor is thought to play a significant role in the development of CPL, although it could not be identified to date. This genetic predisposition may be present in any of the elements of the vicious cycle.

Despite numerous attempts to unravel the etiology and genetic origin of CPL, the causative factor and subsequent pathogenesis of this disease remain unknown. Two diverging hypotheses have been proposed, and although the causative factors in these hypotheses are rather divergent, several mutual elements can be identified. Dysfunction of the lymphatic system, fibrosis, inflammation, and an aberrant elastin network are the four factors that appear to be associated with CPL, and provide the basis for established hypotheses on the pathogenesis. However, the chronology of events within the pathogenesis of CPL proves to be the most debatable factor. For example, lymphedema may drive inflammation and fibrosis, but on the contrary, chronic inflammation and subsequent fibrosis may also adversely affect the functionality of the lymphatic system [21]. As such, a vicious circle can be created whereby these factors might permanently reinforce each other (Figure 2). This is probably the main reason why no initiating or triggering factor for this condition has been identified to date. Consequently, several diverging hypotheses have been formulated regarding the cause of this disorder. However, it is possible that current hypotheses do not stand alone, and each may explain part of the pathogenesis. In addition, a genetic predisposition is assumed to play an important role in the development of CPL, although no specific associated genes have been identified to date. It is also unclear in which factor within this vicious circle a genetic predisposition could be found. For example, alterations in the elastin network could result from an innate lack of elastin quantity or from a genetic predisposition for progressive elastin breakdown. Inflammation could possibly be the result of chronic hypersensitivity or an autoimmune response. In addition, draft horses may be predisposed to develop an excessive accumulation of fibrous connective tissue. Moreover, genetic defects could lead to primary lymphedema. Further research is required to establish both the chronology and the possible genetic origin of the factors leading to the clinical onset of CPL.

### 2.1. Lymphatic Disease

A first hypothesis proposes that a defect in the lymphatic elastic system is the initiating factor of this disease, due to the demonstrated quantitative and qualitative dermal and perilymphatic elastin abnormalities in CPL-affected draft horses [6,17]. Clinically normal draft horses of a susceptible breed were found to have had lower elastin concentrations (based on desmosine concentration in the skin) when compared with horses of a non-susceptible breed. However, this difference was statistically significant only for the mid-dermis of the neck region [22]. No changes were observed in the elastin network of other organs, including the elastin-rich aorta, leading to the hypothesis that CPL is a generalized skin disorder [6,17,22]. Moreover, the presence of anti-elastin antibodies in affected draft horses suggests the pathological degradation of elastic fibers and tissue damage, resulting in a failing elastic-skin network [23]. These lower quantities of dermal elastin and the dysfunction of the elastin matrix may result in an inadequate lymphatic elastic support, leading to reduced lymphatic drainage [6,17,22]. The elastin network fulfils an essential role in facilitating lymph clearance since elastic fibers interconnect lymph vessels and various dermal structures, forming a ‘path of low resistance’, and promoting the uptake of fluid and molecules into the initial lymphatics [24]. In clinically affected draft horses, a delayed lymphatic clearance indeed has been demonstrated, and the extent of this delay was found to have been correlated with the severity of the clinical signs [7]. The final stage of lymphedema is characterized by a complete lymph stasis as a consequence of intrinsic lymphatic dysfunction, with severe vessel dilatation and tortuosity, as observed in the final stages of CPL [6]. Lymphedema can also promote extracellular matrix deposition and fibrosis of the tissues, causing structural changes that can manifest as fibrous nodules, thickened skinfolds, and even deformation of the distal limbs [6,8,25,26]. In this hypothesis, CPL in draft horses is considered to be a primary type of lymphedema, mainly since no causes of secondary lymphedema (e.g., lymphatic obstruction, filariasis, or traumatic injury) have yet been demonstrated in affected breeds [6,8,17,22]. The frequent occurrence of secondary infections in CPL-affected horses is attributed to an impaired circulation of lymph fluid and lymph drainage, which results in an impaired skin-barrier function, a compromised function of the skin immune system, and an accumulation of peripheral tissue antigens [10,27]. Under this hypothesis, genetic research has been established to evaluate potential candidate genes, such as the Forkhead box protein C2 (FOXC2), which is associated with primary lymphedema (lymphedema–distichiasis syndrome) in humans and the elastin gene (ELN), but no aberrations have yet been found in draft horses [8,28].

### 2.2. Inflammatory Skin Disorder

A second hypothesis proposes that chronic inflammation is the primary mechanism underlying the pathologic changes of the distal limbs in CPL-affected horses. This inflammation initiates tissue edema and lesions that progress from scaling to hyperkeratosis and hyperplastic fibrosis. Various causes such as bacterial infections, mange infestation, vasculitis, and contact with chemical irritants could trigger inflammation, leading to the clinical onset of CPL [18,29,30,31]. Chronic inflammation has been reported to drive lymphangiogenesis, resulting in a dysfunctional formation of lymph vessels and causing an inflammation-induced contractile dysfunction [32]. This, in turn, may cause decreased drainage of lymph fluids, lipids, and immune cells, resulting in a persistent inflammation that hampers skin regeneration and possibly triggers an exaggerated (auto)immune response [11]. Moreover, elastase secretion from neutrophils, a known process in a variety of chronic inflammatory diseases, again results in pathological degradation of elastin in the skin, as observed in affected draft horses [11,23]. From this perspective, a second hypothesis was formulated suggesting that CPL is an overregulated inflammatory (auto)immune response, and chronic inflammation is the main cause of pathological changes in CPL-affected horses [6,10,11,17,18,22,30,31,33]. François was able to demonstrate the involvement of the immune-response processes in the CPL phenotype by pathway-based analysis using genomic regions that were significantly associated with CPL in a genome-wide association study [10]. In addition, Mittmann et al. had previously identified several candidate genes under the hypothesis of an overregulated inflammatory autoimmune response, two of which were confirmed to lie within regions associated with CPL (ubiquitin protein ligase E3A and CD109 on ECA1 and ECA10, respectively) [10,11]. Further functional research is needed to identify the genetic cause underlying CPL and the involvement of the immune system [34].

## 3. Lesions and Clinical Signs

### 3.1. Primary CPL Lesions

Clinical signs may manifest at any age, although lesions typically develop in animals starting at two years of age [6]. From the onset of the first clinical signs, full recovery is currently unattainable, and the clinical signs gradually worsen over time. However, the speed of progression is largely dependent on additional influencing factors such as hygiene, skin care, and the treatment of secondary infections [5]. 

**Figure 3:**
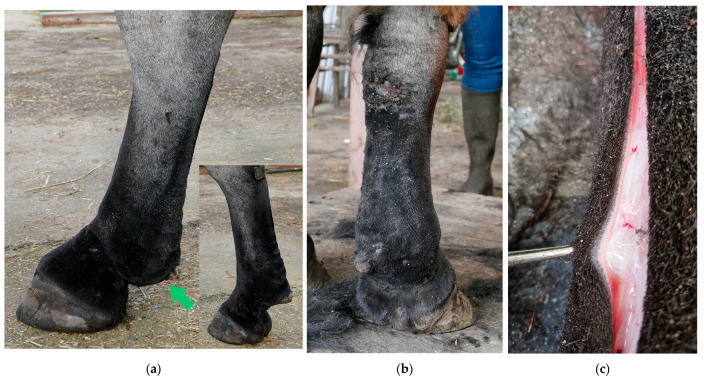
Dermal and subcutaneous edema of the distal limbs. (**a**) In the early stages of CPL, localized onset of ‘pitting’ edema can be observed in the fetlock and pastern regions (green arrow). In this horse, slight folding of the skin on the plantar aspect of the cannon bone is also visible. Insert, contralateral leg with absence of edema. (**b**) Generalized edema of the distal right forelimb. Notice skin fissures on the carpus. (**c**) Subcutaneous and dermal edema at the level of the palmar region of the distal forelimb in a yearling draft horse.

Edema is mainly characteristic of the early stage of CPL and gradually progresses to dermal fibrosis [5,8]. Therefore, edema is often only observed in young animals. It is presented by thickened skin in the pastern and fetlock region, also referred to as “pitting edema” (Figure 3). The edema usually passes unnoticed since it is masked by the heavy feathering in draft horses.

**Figure 4:**
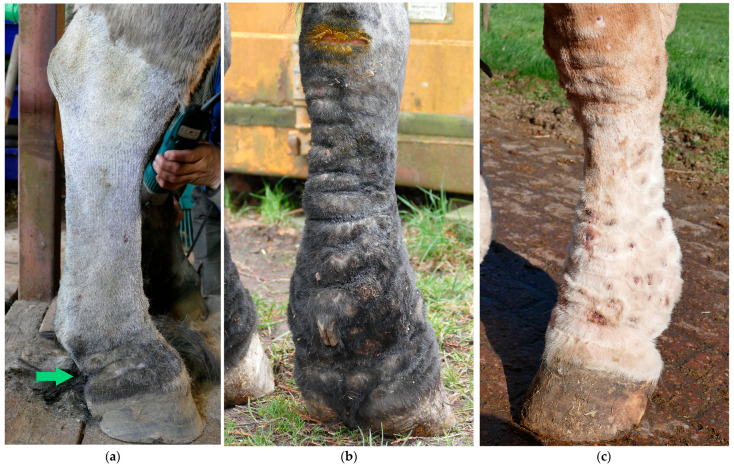
Skinfolds, the main feature of CPL in the Belgian draft horse. (**a**) Early-stage lesion of CPL: two folds in the pastern region (green arrow). (**b**) Advanced stage of CPL: expansion of the folds to the palmar aspect of the cannon bone. (**c**) Final stage of CPL: skinfolds appear dorsally on the distal limb.

The progressive development of skinfolds is the most distinctive clinical sign of CPL (Figure 4). Initially, dermal thickening with the formation of skinfolds in the pastern region occurs. In more advanced stages of the disease, these lesions gradually extend to the palmar, the plantar aspects of the fetlock, and the cannon bone. In the most severe and final stages of CPL, skinfolds may also appear dorsally, circularly enclosing the entire leg. At the joints, the dermal lesions can limit the horse’s movement and even cause lameness due to the inappropriate alignment of the articular bones. In addition, appositional skin of large folds is subject to friction of movement, increased local heat, maceration from retained moisture, and irritation from the accumulation of debris, causing a superficial inflammatory dermatosis or intertrigo [35].

**Figure 5:**
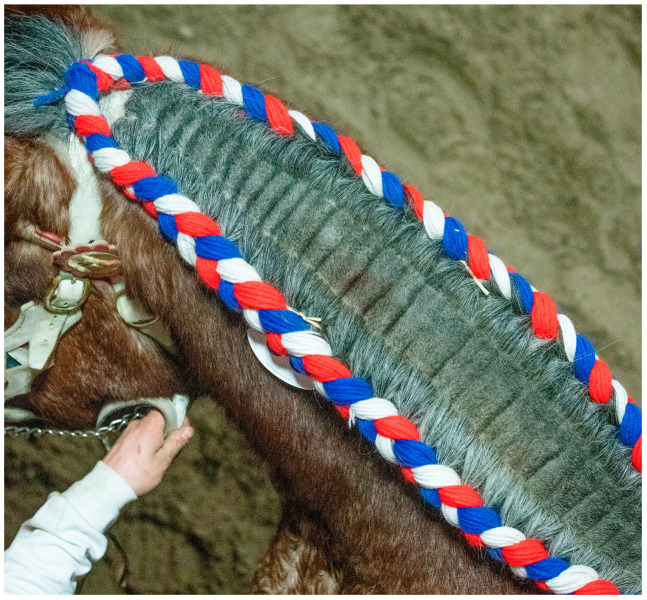
Skinfolds in the neck of a Belgian draft horse. A 6-year-old Belgian draft horse stallion exhibiting folding of the skin in the neck region. The mane has been partially clipped to facilitate easier braiding during studbook inspections (Courtesy of T. van der Weerden, 2021).

It was hypothesized that CPL may be a generalized skin disorder, affecting not only the dermis of the distal extremities but also the elastin-rich skin of the neck [6,17,22,36]. In older stallions, skinfolds in the neck region appear to be a frequently encountered phenomenon during studbook inspections, suggesting a potential systemic involvement of the condition (T. van der Weerden, personal communication, 2022) (Figure 5). In addition, a case of generalized skinfolds in a Belgian draft horse stallion has been described by De Keyser et al. in which the affected horse displayed folding of the skin not only on the distal limbs but also in the neck and the trunk region [36].

**Figure 6:**
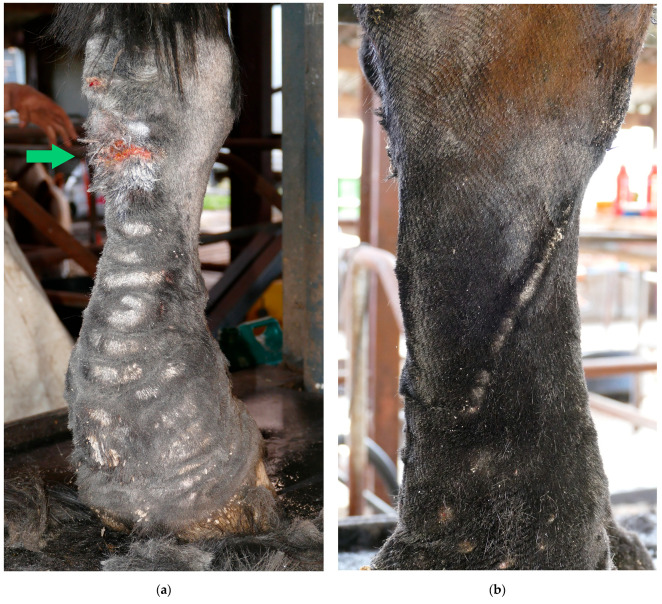
Skin fissures on the carpus and tarsus. (**a**) Typical skin fissure on the palmar aspect of the carpus (green arrow), also referred to as ‘mallenders’. (**b**) A linear scaling and hyperkeratotic lesion on the lateral aspect of the right hind limb, resulting from a persistent exudative lesion on the dorsal aspect of the tarsus (‘sallenders’).

CPL is characterized by hyperkeratosis of the skin on the distal limbs with a high quantity of epidermal keratin, causing the skin to become dry, scaly, and prone to mechanical damage [5,6,36,37]. The tarsus and carpus, especially the dorsal aspect of the hock and the palmar aspect of the carpus, where skin is subject to high levels of mechanical stress when the horse moves, seem to be susceptible to hyperkeratotic lesions. These lesions may result in skin fissures, known as ‘mallenders’ (the palmar aspect of the carpus) and ‘sallenders’ (the dorsal aspect of the hock) (Figure 6) [38]. Without proper management, the skin underlying these scaly patches will become ulcerated, painful, and prone to secondary bacterial infections. Moreover, when infected, the lesions on the dorsal aspect of the hock often produce a moist exudate that runs down the lateral limb side, resulting in crusty, thickened lines on the lateral aspect of the hind limbs [36].

### 3.2. Lesions Associated with CPL

**Figure 7:**
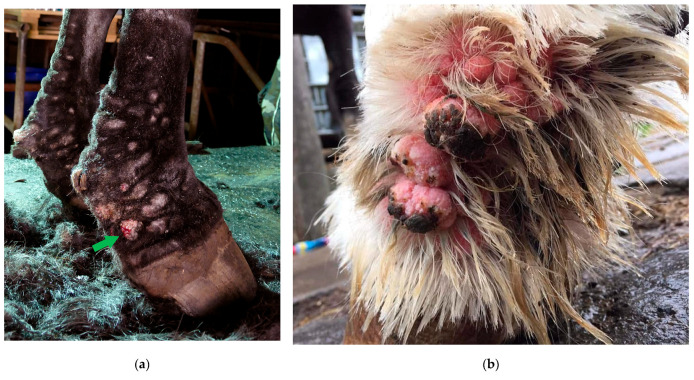
Verrucous pastern dermatitis. (**a**) Wart-like nodules (green arrow) in the pastern region of a Belgian draft horse mare. (**b**) The skin in these areas is highly sensitive to trauma, and consequently poses a high risk of infection and necrosis (Courtesy of F. May, 2021).

Dermatitis verrucosa is a chronic hyperplastic form of pastern dermatitis, characterized by hairless, wart-like elevations and sclerosis of the skin, and hypodermis on the distal limbs, mainly affecting draft horses (Figure 7) [29]. These wart-like lesions are marked by papillomatous or polyploid tissue with epidermal hyperplasia, marked hydropic change, and parakeratosis of the stratum corneum [39]. This clinical presentation of pastern dermatitis is often referred to as a clinical sign of CPL based on the similarities with the classic signs of CPL, i.e., the predilection for cold-blooded horses with abundant feathering on the legs and the progressive nature of the lesions [5,29,39,40]. However, some notable differences can be observed: the wart-like lesions are not always present in horses with CPL (unlike the skinfolds) and, if present, often do not affect all four limbs [41]. Both the etiology of these lesions and whether verrucous dermatitis is a separate clinical identity in draft horses or an atypical clinical presentation of CPL remain unclear to date.

**Figure 8:**
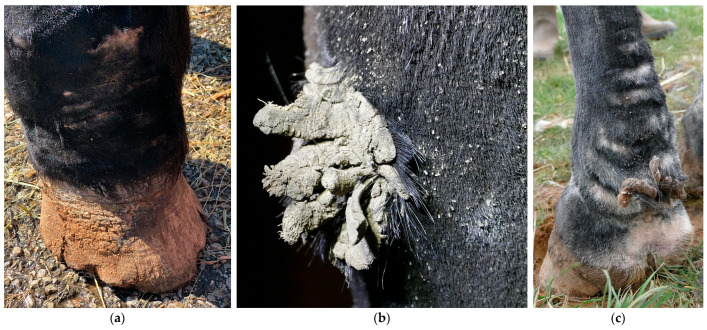
Poor hoof horn quality and prominent chestnuts and ergots in draft horses with CPL. (**a**) The hoof wall exhibits poor keratinization, with visible signs of proliferation, scaling and rings of hoof growth (Courtesy of D. Conder Emge, 2021) (**b**) Hyperplastic and hyperkeratotic chestnut and (**c**) ergot in Belgian draft horses.

Hoof quality is significantly correlated with the severity of CPL lesions in draft horses [33]. Since the hooves are brittle and easily chipped and cracked, hoof abscesses are frequently encountered in draft horses with CPL [5]. Infrequent and poor grooming of the hooves does not influence the occurrence or severity of the hoof-horn deterioration [18]. In addition, the prominence of chestnuts and ergots has been shown to be significantly correlated with the severity of CPL (Figure 8). However, it is not clear if these features are consequences of CPL rather than risk factors for the disease’s severity [9]. The clinical presentation of the poor hoof-horn quality, and the prominence of ergots and chestnuts is remarkably similar to that of coronary band dystrophy, a condition most often described in draft horses. In equine coronary band dystrophy, similarly to CPL-affected skin, a marked proliferation and hyperkeratosis of the epidermis of the coronary band is present, and in some cases, chestnuts and ergots [42,43,44]. Like CPL, equine coronary band dystrophy is a chronic condition of unknown etiology and pathogenesis, and especially draft horses are considered predisposed [44,45]. Hoof canker (chronic equine proliferative pododermatitis) is a second clinical condition affecting the hoof, ergots, and chestnuts, that is considered to be prevalent in these breeds as well [46,47,48,49]. To date, it is not known whether both conditions are related to the occurrence of CPL, and whether the poor hoof quality in horses that are affected by CPL is a consequence of the presence of these two conditions or arises from a separate etiology. However, a correlation between CPL and hoof canker has been suggested in the literature [50,51].

### 3.3. Secondary CPL Lesions

**Figure 9:**
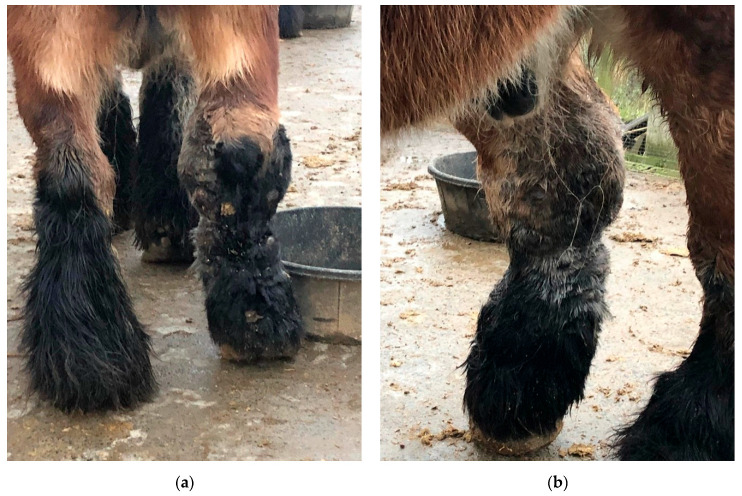
Cellulitis/lymphangitis in a Belgian draft horse with CPL. (**a**) Plantar view and (**b**) medial view of the right hind limb of a CPL-affected draft horse, displaying a ‘stovepipe’ appearance, which can be observed in both lymphangitis and cellulitis (Courtesy of A. Callens, 2022).

Because of the pathological changes of the skin and lymphatic system, the skin barrier, and consequently the skin immunity, will be hampered, leading to an increased susceptibility to secondary infections in CPL-affected draft horses [5]. Skin lesions provide a port of entry for bacteria, often resulting in an acute onset of cellulitis or lymphangitis. Cellulitis is defined as a diffuse bacterial infection of the dermis and subcutaneous tissues [52], while lymphangitis is a bacterial infection of the lymphatic vessels in the limb [53]. Lymphangitis may accompany cellulitis and vice versa [54]. In both conditions, the limb may become warm, painful, and will have a ‘stovepipe’ appearance as a result of the significant swelling (Figure 9). Since the clinical presentation of lymphangitis is almost identical to cellulitis, it may be challenging to differentiate with certainty between the two, based on clinical signs only [55]. As a result, both terms are often used interchangeably in practice [56]. It is essential to note that lymphangitis or cellulitis may occur in the absence of CPL, although CPL-affected horses are more susceptible to developing these conditions.

**Figure 10:**
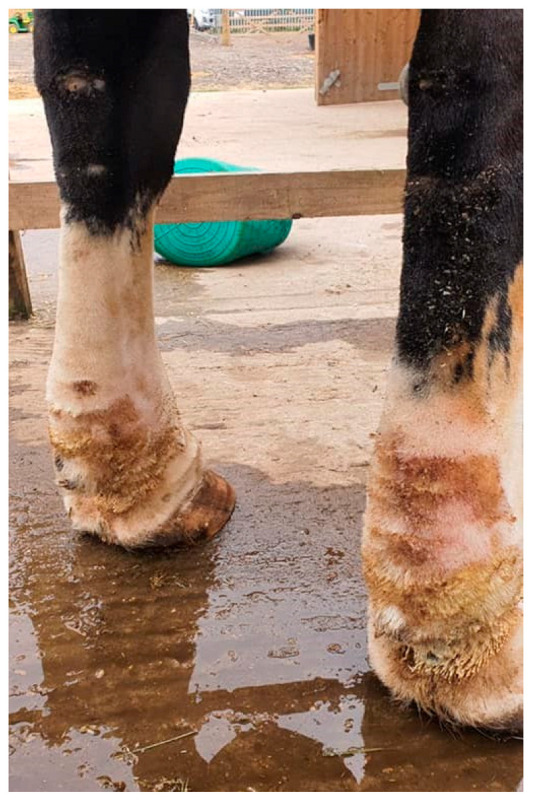
Superficial pyoderma in a horse with CPL. The skin displays characteristic signs of bacterial infection, including inflammation, erythema, and exudate (Courtesy of B. Young, 2021).

Bacterial infections of the superficial skin (superficial pyoderma) occur frequently in horses affected by CPL, as the skin is more sensitive to trauma, causing easy development of skin lesions. Moreover, the feathering on the legs facilitates moisture retention. In addition, lymphatic dysfunction results in a lowered skin immunity, facilitating bacterial invasion and infection or pastern dermatitis [5,57]. In horses with CPL, the opportunistic pathogen *Staphylococcus aureus* is the most prevalent bacterial species isolated from skin samples [5,58], as in warmblood horses with pastern dermatitis [59]. Affected skin is irritated, red, inflamed, and/or swollen, and will develop a discharge that appears ‘greasy’ or sticky (Figure 10). The fungal infections most commonly reported in horses are dermatophytoses and onychomycoses [60].

**Figure 11:**
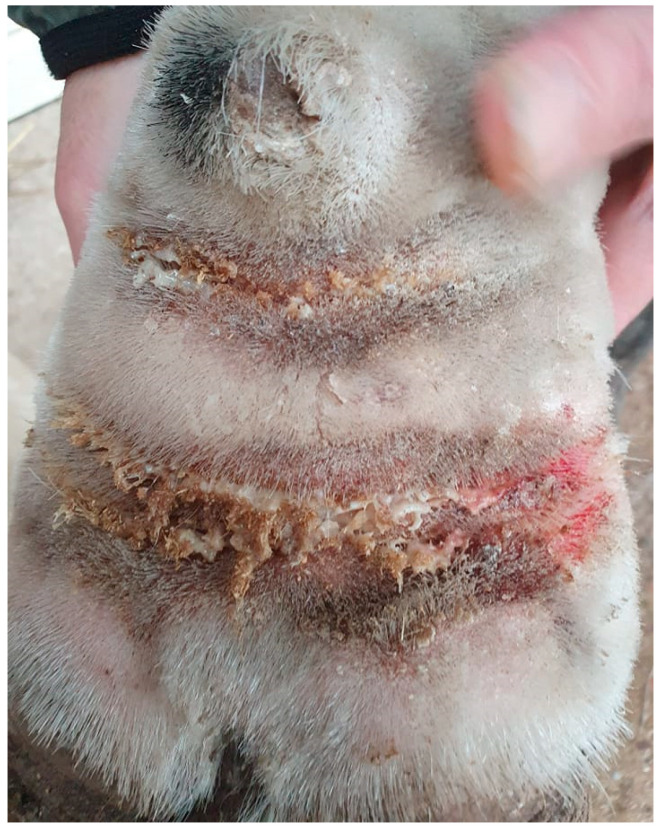
Intertrigo or skinfold dermatitis in a CPL-affected horse. The skin between the folds is ulcerated, erythematous, and covered with a serous to suppurative exudate (Courtesy of V. Clifton, 2022).

In advanced stages of CPL, severe intertrigo can develop between large skinfolds. This is characterized by the dissolution of the stratum corneum, exudation with oozing, secondary bacterial and/or yeast infections, and, in severe cases, necrosis (Figure 11). A foul odor may be present, and the horse may react painfully when touched in this area. Moreover, these lesions create an ideal niche for maggot infestation.

**Figure 12:**
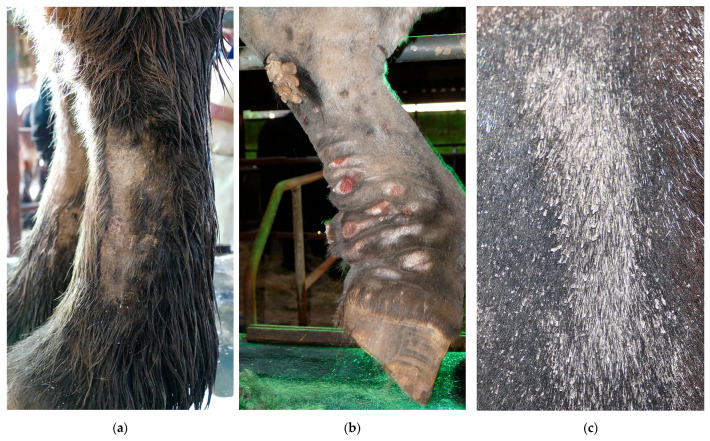
Clinical signs of mange in draft horses. (**a**) Alopecia is a characteristic feature of mite infestation, commonly observed on the lateral or medial aspect of the cannon bone. (**b**) Due to intense pruritus, the horse may exacerbate the condition by vigorously scratching or rubbing the affected area, leading to self-induced wounds on the medial side of both the front and hind limbs. (**c**) An extremely scaly patch of skin as a result of a severe mite infestation. Note the typical white debris surrounding the hair shafts.

In addition to bacterial infections, parasitic infestations are a significant secondary complication in horses with CPL. A common parasitic infestation that is found in affected horses is mange, caused by *Chorioptes bovis* [5,29]. These mites may present an aggravating factor or a concurrent complicating factor. Draft horses may be more susceptible to chorioptic mange due to the presence of heavy feathering that traps skin scales and epidermal debris, providing a rich and necessary food supply for mites, while also protecting them against extreme temperatures. Chorioptic mange causes pruritus, lichenification of the skin, alopecia, scaling, and crusting of the distal limbs (Figure 12). As a result of the mite infestation, the horse may become restless and frequently stamp its legs, eventually leading to auto-mutilation. A second parasitic infestation that is often prevalent in severely affected draft horses, especially during the summer, is a maggot infestation. Flies (e.g., *Lucilia sericata*) are attracted to the warm and moist environment between prominent skinfolds, where fly larvae develop and feed on dead tissue and wound debris.

### 3.4. Histopathology

**Figure 13:**
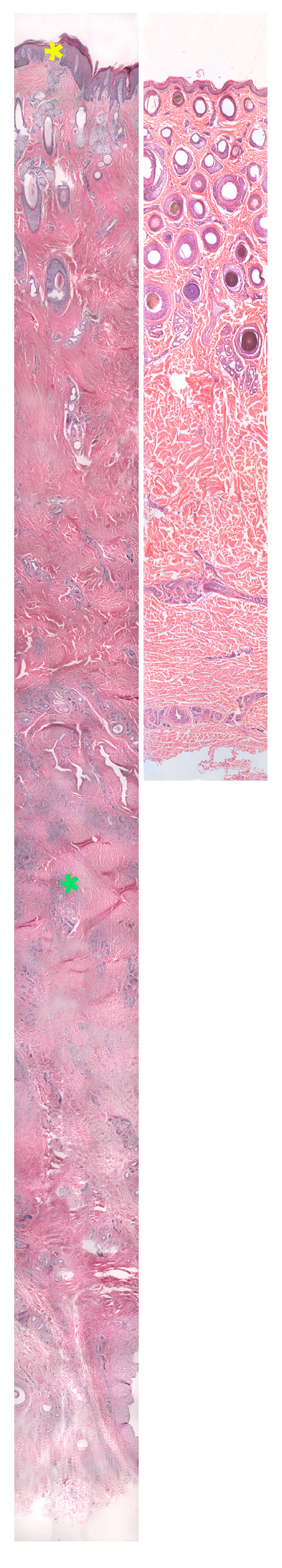
Histology of the skin from the pastern region of a CPL-affected Belgian draft horse and a non-affected Belgian draft horse, both aged 15 years (hematoxylin and eosin, magnification ×12.5). The skin of a CPL-affected draft horse (**left**) is characterized by hyperplasia of the epidermis and hyperkeratosis (yellow asterisk). In the dermis, extensive fibrosis is present (green asterisk). The overall thickness of the skin is significantly increased compared with the skin of a non-affected Belgian draft horse (**right**).

The descriptions of histological lesions of CPL are limited to the chronic stages of the disease. The main characteristics of these lesions include marked hyperkeratosis, hyperplasia of the epidermis with the formation of irregular rete ridges, excessive dermal fibrosis, and the presence of a perivascular dermatitis predominated by lymphocytes (Figure 13) [6,33]. Mainly the alterations in the vasculature at the level of the dermis and epidermis have been described. The lymphatic vessels of the skin exhibit the most significant histological changes, whereby in mildly affected horses, the lymphatics are mildly-to-moderately dilated. As lesions aggravate, these vessels become even more dilated and tortuous. Perilymphatic fibrosis is also often noted, varying from thin, indistinct layers to thick, prominent layers of collagen bundles arranged circumferentially around the lymphatics [6]. CPL-affected horses with mild lesions may display proliferation of the middle and deep vascular plexi of the skin, with smooth muscle hyperplasia [5]. In more advanced stages, a prominent proliferation of the superficial vascular plexus can be observed [6].

In severely affected limbs, the elastic layer of the subepidermal dermis and the concentric ring of elastin fibers surrounding the lymphatics of the deep dermis are markedly distorted and separated by fibrous tissue [5]. In parallel with the clinical CPL progression, the initial low dermal elastin quantity in affected horses significantly increases; however, the elastin is assembled in a disorganized and non-functional form [22]. In contrast, the perilymphatic elastin is histologically sparse in severely affected horses [6].

## 4. Factors Associated with Occurrence and Severity of CPL

CPL is assumed to be a multifactorial disorder [10,18,61]. This implies that its variation in both the disease susceptibility and the development of clinical signs are determined by an interplay of two factors: the genetic variance of an individual (genotype) and the environmental influences. Although no genetic traits associated with CPL have been identified in affected horses, a limited number of studies have identified environmental risk factors for CPL occurrence and the disease severity [9,13,18,33,61,62]. 

### 4.1. Age

Age has been found to be significantly correlated with disease severity [9,33]. CPL is a progressive disease and therefore clinical signs will deteriorate with increasing age. De Keyser et al. demonstrated in a population of 980 Belgian draft horses that only 14.00% yearlings showed mild clinical signs, whereas 85.86% of a subset of horses older than 3 years was clinically affected [9]. The severity of lesions, especially the skinfold thickness, increases for both mares and stallions from the age of 3 years onwards [9]. Sievers and Distl also confirmed that the first onset of clinical signs starts in horses aged 1–2 years [62]. The disease progression ceases at a mean age of 16 years in stallions, 18 years in geldings, and 20 years in mares [62].

### 4.2. Gender

Sex, as well, is significantly associated with the occurrence of lesions, with the disease prevalence being significantly higher in stallions compared with mares [18]. Indeed, Sievers and Distl reported that males have a fivefold higher risk of developing CPL lesions compared with females [62]. Furthermore, De Cock et al. and Verschooten et al. have reported that, in general, stallions are more severely affected than mares [6,63]. However, sex was not correlated with disease severity in a study by Geburek et al. [33]. Although lesions appear sooner in mares compared with stallions, disease progression is more rapid in stallions [9,62]. The latter may explain the difference in age at which the disease’s progression stabilizes. The difference in the disease’s progression in stallions compared with mares can be attributed to differences in housing and less access to pastures, as well as feeding regimes and more selective breeding [8,62].

### 4.3. Secondary Infections

CPL-affected draft horses are more susceptible to secondary dermal infections and pastern dermatitis, caused by bacteria, fungi, or parasites, as the local skin immunity is impaired [64]. Conversely, these secondary infections may aggravate CPL progression and complicate CPL diagnosis [6,18,29]. In draft horses, the prevalence of chorioptic mange ranges from 50% up to 95% [6,29,33,65,66]. This predisposition could be explained by the presence of heavy feathering [67]. *Chorioptes bovis* infestation may affect the progression of CPL in draft horses, manifesting with edema, lichenification, and excessive skinfolds that can progress to verruciform lesions [29,66]. In addition, the consequent inflammatory response may possibly trigger the onset of CPL. Therefore, addressing secondary infections is crucial in the supportive treatment of CPL-affected horses [14].

### 4.4. Housing

According to Geburek et al., horses kept in outdoor pens on rubber flooring are significantly less-severely affected than those kept on sand or soil [33]. Similarly, Sievers and Distl found that horses kept on pastures and paddocks had lower CPL scores [62]. In addition, poor stable hygiene significantly increases CPL severity [33,62]. This could be explained by the link between poor hygiene and the prevalence of pathogens that can cause secondary infections [40].

### 4.5. Intended Use of the Horse

In horses kept for breeding and production of meat and/or milk, the lesions are statistically and significantly more prevalent than in horses that are used for riding and working (traditional agriculture), although neither is significantly correlated with the severity of clinical signs [18,33]. Sievers and Distl also failed to demonstrate a significant correlation between different types of horse use and their work, and CPL severity [62]. 

### 4.6. Diet

Feeding regimes that include silage in winter have been shown to increase CPL prevalence and severity in German draft horses [18,62]. In contrast, a diet of hay and straw has been found to lower the risk of high CPL scores [62]. Feeding concentrate has also been found to increase CPL severity [62]. Moreover, Geburek et al. concluded that the amount of maize and oats in the diet of horses may influence the clinical degree of the disease’s severity [33]. De Keyser has reported that stallions are frequently given an energy-rich diet in preparation for the annual stallion selections, which could possibly explain the observed higher disease prevalence and severity in stallions compared with mares, as demonstrated by De Cock et al., and Verschooten et al. [6,8,63]. However, this difference in clinical severity might as well be attributed to gender and hormonal differences. Although the exact mechanism connecting a diet that is high in sugar and starch, and the progression of clinical signs of CPL has not yet been fully elucidated, several studies have demonstrated that starch has a direct influence on the inflammatory response in the body and the level of pro-inflammatory cytokines in the bloodstream. Therefore, sugar and starch are likely to contribute to the inflammatory state of horses with CPL, which are already prone to inflammation at the level of the distal limbs. High-starch diets elevate plasma concentrations of interleukin-1β (IL-1β) as soon as one hour post-eating [68]. This increase in IL-1β is possibly due to changes in the intestinal pH that result from the rapid bacterial fermentation of starches and sugars in the digestive tract [68]. Moreover, a higher level of tumor necrosis factor-α is observed in horses that are fed a diet high in sugar and starch, indicating an increase in some level of systemic inflammation [69]. In conclusion, feeding management with restricted or no concentrates and hay silage reduces the risk of developing severe CPL lesions [16,62]. However, further research into the influence of nutritional parameters is required to determine the exact effect of the diet on the course and progression of CPL.

### 4.7. Features of the Distal Limbs

The cannon bone circumference, the prominence of feathering, ergots, chestnuts, and bulges in the fetlock region are significantly correlated to the disease’s severity [9]. However, it remains unclear whether these features are consequences of CPL or if they are risk factors for the disease’s severity. Additionally, the hoof quality is also significantly correlated to the severity of CPL [33]. In a study conducted by Wallraf on German draft horse breeds, limb hair characteristics (e.g., hair implantation density) were found to have been correlated with the disease’s prevalence [18]. Another study revealed a correlation between the presence of skin without pigment (white markings) and the clinical severity of CPL lesions [13], although Geburek et al. failed to confirm this correlation [33]. Federici et al. demonstrated that horses with more pronounced white markings had an increased risk of suffering from pastern dermatitis, sunburns, and hoof-horn abnormalities [70]. However, a specific correlation between CPL and white markings has not yet been established.

## 5. Diagnosis

### 5.1. Clinical Examination and Scoring

The observation of its unique clinical presentation (e.g., skinfolds, nodules on the distal legs) in a susceptible breed is currently the most frequently used method to establish the diagnosis of CPL. However, a definitive diagnosis cannot be made based solely on a visual clinical examination. Further analyses are required to confirm the diagnosis by ruling out other primary causes of pastern dermatitis (e.g., fungi, bacteria), or to determine whether these pathogens are secondary complications of CPL. Since the clinical signs in the early stages of CPL may be rather subtle, palpation is necessary for diagnosis. In addition, clipping of the feathering is strongly recommended as it enhances palpation and visual inspection.

A guideline to categorize the clinical evaluation of CPL in Belgian draft horses was first developed by Colman and De Keyser (Table 1), and is now widely used to score CPL lesions and their severity [71]. Subsequently, similar scoring systems were established by Affolter and De Keyser, but are currently not being used in practice [5,36]. The current scoring system is based on visual inspection (skin surface and feathering) and palpation (swelling and deformation) of the distal legs. Each leg is assigned a letter score in one of five scoring classes (AA, A, B, C, and D). The score AA refers to normal legs without any indication of CPL impairment, while D indicates the presence of extremely severe CPL lesions. However, this scoring system has several limitations. For instance, it includes secondary lesions such as skin lesions and hair consistency as criteria, which do not always correspond to the extent of the primary lesions (skin thickening). Skin lesions, such as wounds and exudate, are often the result of secondary infections that may complicate CPL [6,18,29], but they do not necessarily correlate with the severity of CPL. CPL is currently incurable, but secondary lesions can be resolved through medical care. The same applies to the condition of the feathering. Rough and broken hairs are often seen in horses with mite infestations, resulting in pruritus and leg abrasions, and consequently affecting the quality of hairs on the lower legs [29,67,72]. This complicates using the condition of the hairs as a parameter in CPL assessments. Moreover, there appears to be a practical need for refinement and additional differentiation within the current broad categories and grades of CPL.

**Figure 14:**
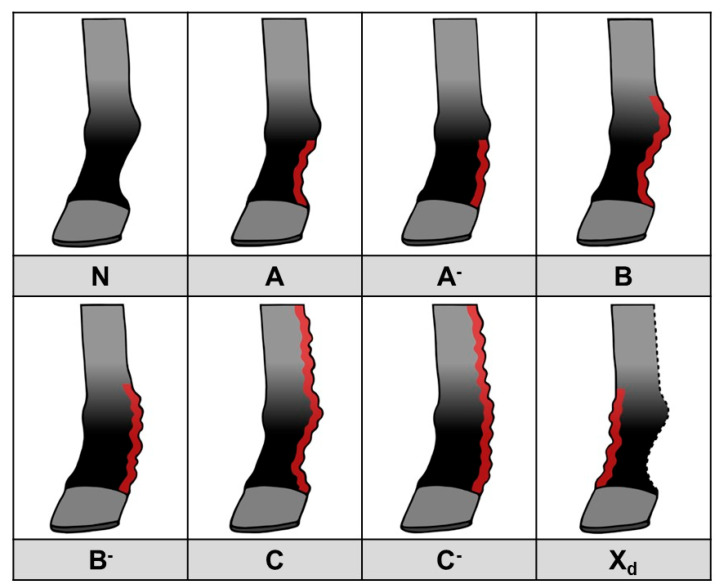
CPL scoring system, based on the presence and distribution of skinfolds and nodules. N: Normal limb, no clinical signs of CPL. A: Mild to moderate skinfolds and/or nodules in the pastern region, below the fetlock, in which the outline of the pastern maintains a concave shape. A^-^: Thick skinfolds and/or nodules in the pastern region, below the fetlock, in which the pastern cavity is completely filled with skinfolds and/or nodules. B: Mild to moderate skinfolds and/or nodules in the pastern region, including the fetlock, in which the outline of the pastern maintains a concave shape. B^-^: Thick skinfolds and/or nodules in the pastern region, including the fetlock, in which the pastern cavity is completely filled with skinfolds and/or nodules. C: Mild to moderate skinfolds and/or nodules in the pastern, fetlock, and metacarpal/metatarsal region, in which the outline of the pastern maintains a concave shape. C^-^: Thick skinfolds and/or nodules in the pastern, fetlock, and metacarpal/metatarsal region, in which the pastern cavity is completely filled with skinfolds and/or nodules. X_d_: Presence of skinfolds and/or nodules on the dorsal side of the limb. This can be present in any of the previously mentioned scores, resulting in A_d_^(-)^, B_d_^(-)^, or C_d_^(-)^.

Therefore, we have developed an updated scoring system that is more practically applicable and takes into account only the primary features of CPL (Figure 14). Here, a score is used to indicate both the location (distal, proximal, palmar/plantar or dorsal) of skinfolds and nodules, as well as the dimension (depth) of the present skinfolds. Lesions that may be related to secondary infections, such as wounds, exudate, and rough and broken hairs, can be assessed separately. This scoring system can be used by veterinarians and horse owners for objective communication about CPL lesions. Furthermore, it can be used as a descriptive tool to classify CPL severity in horses, and to allow the simple evaluation of progression of the lesions. This new method of scoring is based on palpation of the distal limbs, but it can also be applied when assessing radiographic images, as the same parameters can be evaluated. This offers significant advantages since palpation of the distal limbs is often hindered by the presence of abundant feathering. In such cases, radiographic imaging can visually substantiate the scoring.

### 5.2. Punch Biopsy

Most diagnostic morphologic lesions are typically observed in the deep dermis and subcutis. Thus, regular skin punch biopsies that only sample the superficial dermis may not always be useful [5]. Therefore, a double-punch biopsy technique is recommended to visualize the changes of the deeper lymphatic vessels and vasculature. A double-punch biopsy can be obtained by using an 8 mm punch through superficial and mid-dermal epidermis, followed by a 6 mm punch through the previous 8 mm biopsy site to harvest the deep dermis and subcutis [5]. However, in CPL-affected horses, a delayed wound healing and an increased risk of secondary infection may be cited as contraindications for a double-punch biopsy.

### 5.3. Medical Imaging

**Figure 15:**
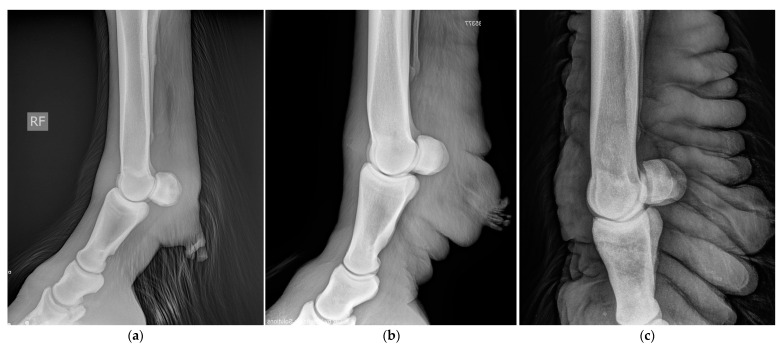
Radiographic images of the distal legs in CPL-affected horses. (**a**) A non-affected Belgian draft horse, displaying no skinfolds. (Courtesy of M. Oosterlinck and K. Vanderperren, 2023). (**b**) A moderately affected Gypsy Cob mare with clear skinfolds in the pastern region and some slight folding of the skin plantar to the cannon bone. (Courtesy of E. Evrard, 2021) (**c**) A severely affected draft horse with pronounced, thick skinfolds on the overall distal limb. (Courtesy of Boschhoven paard, 2019).

In general, radiographic imaging is considered the most important medical imaging modality for the routine diagnosis of CPL. This technique enables the early detection of skin alterations and provides an important added value in visualizing skinfolds and nodules when palpation is hampered by the presence of abundant feathering on the limbs (Figure 15) [8]. Indirect lymphangiography is a non-invasive technique in which a contrast medium is injected intradermally, followed by radiography to examine the morphology of lymphatic vessels. De Cock et al. have successfully used this technique in draft horses with severe CPL to visualize the tortuous and dilated lymphatic vessels in the distal limbs [6]. In addition, De Cock et al. have demonstrated the presence and severity of lymphatic stasis in CPL-affected draft horses by using lymphoscintigraphy [7]. While lymphoscintigraphy is the method of choice to distinguish lymphedema from other causes of distal-limb swelling in humans, this technique is not routinely used in draft horses.

### 5.4. Anti-Elastin Antibodies ELISA

The breakdown of elastin generates elastin-derived peptides that can be identified by immunocompetent cells, which produce blood anti-elastin antibodies (AEAb). The degradation of elastin in CPL-susceptible breeds has been suggested [17,22]. Indeed, Van Brantegem et al. demonstrated significantly higher AEAb levels in clinically affected draft horses compared with non-affected horses of a susceptible breed and Belgian Warmblood horses [23]. Moreover, the AEAb level increased with the disease’s severity in different clinical groups. Therefore, this ELISA has been proposed as a diagnostic aid for CPL in combination with a clinical examination [73]. However, De Keyser et al. demonstrated a very low diagnostic accuracy of AEAb with the reported ELISA for the diagnosis of CPL, due to a large overlap between the various clinical groups and an association of AEAb levels with the executive lab and the date of blood sampling [74]. Hence, this methodology is highly suggestive of having no use as a diagnostic aid in individual, clinically examined CPL-susceptible draft horses [74].

### 5.5. Genetic Assays

Although CPL is considered a hereditary disease, currently there is no genetic test that is available to diagnose this condition. None of the conducted studies have identified a specific genetic marker and/or mutation that is correlated with CPL. Different candidate genes have been suggested, but further functional research is needed to identify the genetic cause underlying CPL [34].

## 6. Symptomatic Treatment and Management

At present, there is no curative treatment available for CPL. However, careful management and supportive therapy can improve the quality of life of affected horses. These treatments are labor-intensive and must be implemented throughout the horse’s entire lifespan to minimize discomfort and slow down the progression of the disease. Treatment is primarily focused on wound and skin care, preventing secondary infections, and controlling tissue edema.

### 6.1. General Skin Care

In order to prevent secondary infections, accurate hygiene measures must be applied to the predisposed areas. Clipping the feathering is highly recommended for cleaning and applying topical treatment. Basic cleaning can be done with lukewarm water and disinfectant soap, after thoroughly brushing out flakes of skin, especially between the deep skinfolds. When excessive crusting or scaling is present, a keratolytic shampoo can be used. Drying the legs thoroughly is essential to prevent the accumulation of moisture, which can be achieved with a towel or blow dryer to access the deep skinfolds. Topical application of keratolytic ointments that are based on urea and salicylic acid is recommended for areas with marked hyperkeratotic crusts [75,76]. At the level of the palmar aspect of the carpus and the dorsal aspect of the tarsus, it is important to maintain skin flexibility to prevent the skin from tearing, causing persistent wounds. Moisturizing ointments may therefore also be important in the management of CPL-affected horses with dry, crusty skin.

### 6.2. Secondary Infections

Good hygiene practices are essential in preventing and treating secondary (bacterial) infections in CPL. Topical antiseptics (e.g., chlorhexidine and povidone iodine) can be used initially to limit the infection. If antiseptics alone are not sufficient and secondary bacterial or fungal infections of the skin persist, the correct antibiotic or antifungal therapy (topical or systemic) must be administered. Since infections of the skin in CPL usually emerge between the deep skinfolds, it is important to evaluate whether topical antibiotics can be adequately applied to the site of interest. To reduce the risk of promoting antimicrobial resistance, a culture-based antimicrobial therapy should be used, especially in horses that have had multiple or recent systemic exposures to antimicrobials in the past, or in which empirical therapy has failed. Since *Staphylococcus aureus* is frequently involved, the antibiotics most commonly used in horses with pastern dermatitis are trimethoprim-potentiated sulfonamides (15 to 30 mg/kg PO for two to three weeks), often in conjunction with topical antibiotic creams or antibacterial shampoos [77,78,79]. In addition, both Silver sulfadiazine and 2% mupirocin can be used for staphylococcal infections because of their ability to easily penetrate the epidermis [77]. In order to prevent the frequent use of antibiotics, a honey ointment can be a very effective and alternative low-cost product for the treatment of wound infections [80,81]. Fungal infections can be treated with azole antifungals (e.g., Enilconazole, Miconazole 1% shampoo). The prompt treatment of skin infections is critical in preventing fly-attraction and avoiding maggot infestations of wounds.

*Chorioptes bovis* mites are some of the most commonly occurring parasitic infestations in draft-horse breeds with abundant feathering on the distal limbs [65]. Currently, in many countries, there are no registered acaricides that are available for the treatment of mange in horses [82]. This leads to the regular off-label use of veterinary drugs intended for other target animal species, or the use of registered products for horses that are not specifically indicated for the treatment of *C. bovis*, often resulting in varying outcomes. In the literature, several studies have evaluated the use and efficacy of different (veterinary) products for the treatment of mange in horses. In horses with CPL, it is important to consider the possibility that non-burrowing *Chorioptes* mites may become inaccessible within skin crusts and thick layers of hyperkeratosis, and therefore, oral and systemic therapies for *C. bovis* in draft horses may be contraindicated. Nevertheless, Osman et al., and Kotb and Abdel-Rady have achieved a total eradication of mites and the resolution of skin lesions with oral moxidectin, oral ivermectin, and subcutaneously administered doramectin [83,84]. However, the latter two studies also included a full environmental treatment with deltamethrin. In contrast, Littlewood et al., Rendle et al., and Rüfenacht et al. did not obtain the total eradication of mites or any improvement in skin lesions with oral ivermectin, subcutaneous administered doramectin, or oral moxidectin, respectively [66,85,86]. In addition to macrocyclic lactones, the efficacy of topical products for the treatment of *C. bovis* has been demonstrated, including a 1% selenium sulphide shampoo combined with an environmental treatment with a broad-spectrum disinfectant (potassium peroxymonosulfate) [87], a topical treatment with 0.05% phoxim solution [88], and a 5% lime-sulphur solution [67]. However, Rendle et al. demonstrated that treatment with 0.25% fipronil topical spray did not result in total eradication of mites nor a reduction in mange-associated lesions [86]. The use of a 7.5% deltamethrin solution resulted in the occurrence of multiple adverse side effects (e.g., discoloration of the coat, severe itching, and dermatitis) [88]. Amitraz, which is often used as an ectoparasitic in farm animals, should never be administered to horses because of its toxic side effects [89,90]. In addition, *C. bovis* has been shown to be capable of surviving off the host for up to 69 days and, therefore, it is important to apply repeated, regular treatment on both the animal and its environment (e.g., stable, grooming places, horse transporters) [91].

### 6.3. Support of Lymph Drainage

Manual lymph drainage (MLD) was first introduced in 1999 by Berens v. Rautenfeld and Rötting. It is now commonly used as a therapeutic technique in the treatment of lymphedema in horses. This technique involves the manual external manipulation of the lymphatic vessels to stimulate the movement of interstitial accumulated proteins and water towards the circulation. The aim is to move the interstitial fluid in a ‘transterritorial’ manner from the affected area to areas with an adequately functioning lymphatic system [5]. This manual therapy can be complemented by the use of compression bandages, which is then referred to as combined decongestive therapy [5]. These bandages differ from classic stable bandages as they provide a gradual pressure distribution (highest pressure distally). Additionally, during movement (light exercise), the bandages have a massaging effect that stimulates lymph drainage [5]. Powell and Affolter demonstrated a reduction of edema and subsequent limb volume in five affected draft horses with a combination of exercise, repeated MLD, and compression bandage therapy [14]. However, the use of these bandages increased the risk of mite proliferation.

### 6.4. Surgical Intervention

**Figure 16:**
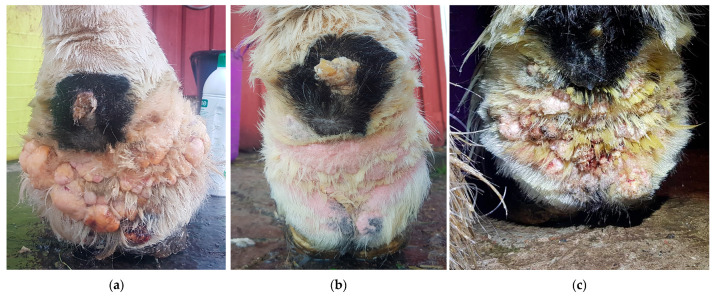
Diathermy surgery in a Gypsy Cob with CPL and warty-like nodules. (**a**) Right distal forelimb before surgery. (**b**) Right distal forelimb 12 days after surgery. (**c**) Regrowth appeared within seven months after surgery. (Courtesy of A. Torstensson, 2022).

**Figure 17:**
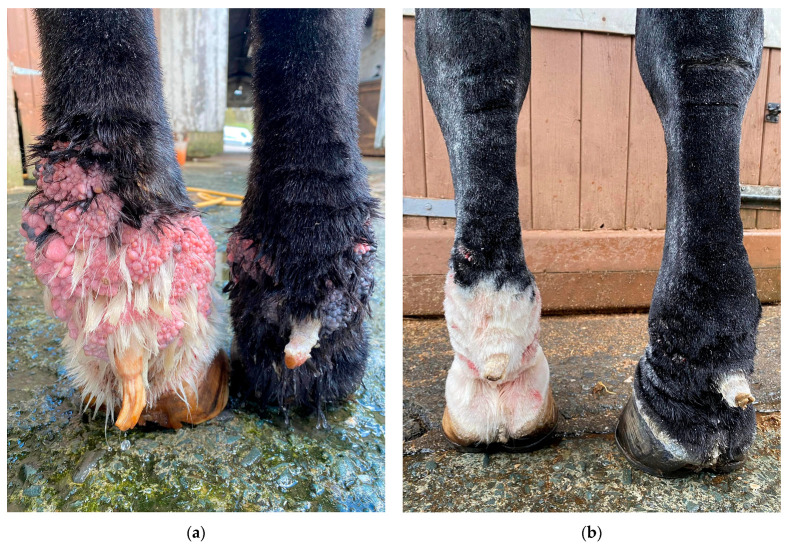
Effects of hemorrhoid cream treatment in a Gypsy Cob with mild CPL and warty-like nodules. (**a**) Palmar view of the forelimbs prior to treatment. Note the presence of a high number of wart-like growths in the pastern region and fetlock. (**b**) Palmar view of the forelimbs after treatment for six months with hemorrhoid cream. The growths have resolved almost completely. (Courtesy of H. Lane and H. Johnsen, 2022).

Although various surgical techniques have yielded positive results, surgical intervention in CPL-affected horses is not highly recommended due to the intensive postoperative supportive treatment that is required, and the risk of developing exuberant granulation tissue in the distal extremities of horses [5,92]. In a Belgian draft horse gelding, dissection and electro-cauterization were utilized to resect several wart-like nodules (verrucous dermatitis) with a favorable postoperative outcome [39]. After a 24-month period, no significant regrowth or complications were observed. In practice, electrosurgery (diathermy) is a second technique that has been employed to reduce the number of wart-like nodules in a Gypsy Cob (Figure 16). However, nodular regrowth was observed within seven months after surgery (personal communication with A. Torstensson, 2022). A third reported technique involves epidermal shaving, which entails removing the hyperkeratotic epidermis and a large portion of nodules and skinfolds [92]. In a Belgian draft horse, this procedure managed to reduce the distal limb diameter but required a rigorous postoperative supportive treatment, including compression bandaging, exercise, wound care, and analgesia, to ensure a long-term effect. To remove wart-like nodules that pose a high risk of trauma and infection, elastic bands, as used in castration by banding, can be utilized as a simple and inexpensive way to remove part of the growths. Remarkably, hemorrhoid cream has recently been used to effectively treat wart-like nodules (Figure 17). This treatment involves the cleaning of the leg with a keratolytic and disinfectant soap, followed by the application of a hemorrhoid cream containing bismuth oxide, bismuth subgallate, zinc oxide, and balsam peru onto the nodules (H. Lane, H. Johnsen, and A. Depuydt, personal communication, 2023). The treatment was applied three times a week during the first month, twice a week for the following three months, and once a week during the last two months. After a period of six months, the majority of the nodules had disappeared.

## 7. Chronic Progressive Lymphedema in Breeding Programs

### 7.1. Integration of a CPL Scoring System

It is widely acknowledged that far-reaching measures are necessary to safeguard the future of CPL-affected draft-horse breeds. Given the high prevalence of CPL within the Belgian draft horse breed and the serious nature of this condition, several approaches have been implemented by the studbooks in an attempt to reduce the prevalence of CPL. Since a genetic susceptibility for CPL is suggested, several studbooks (e.g., KMBT (Belgium) and KVTH (Netherlands)) have integrated a systematic assessment of clinical signs of CPL into their breeding policies. During official contests and stallion studbook approvals of the Belgian draft horse, a veterinarian assesses all four legs (each leg individually) of each horse, using the scoring system described by Colman and De Keyser [71]. In practice, the original scoring system has been adapted by splitting scores A and B into A−/A+ and B−/B+, respectively. This adaptation has been found necessary to adequately differentiate the lesions [93]. Stallions may be presented for studbook approvals at the age of 30 months. During the assessment, the stallion is awarded a total score with a maximum of 30 points, of which 10 points are allocated based on the clinical examination of the horse’s legs. This score must be reassigned annually for a maximum of eight consecutive years. As such, an insufficient leg score can lead to a stallion being disqualified from stud service.

### 7.2. Selection against CPL When Genetic Diversity Is at Risk

For CPL, heritability was estimated at 0.26 (horses > 3 years) and 0.11 (all ages) by De Keyser et al., and 0.14 in a subsequent study by François [10,12]. This is an important indication that environmental factors play a significant role in the pathogenesis of CPL.

Selection against CPL in a breed can be based on phenotypes or estimated breeding values. Currently, selection against CPL in Belgian draft horses is based on phenotype, but this approach has failed to significantly reduce the prevalence of CPL [10]. There are several underlying causes that could explain this unsatisfactory outcome. Most important, CPL has a low-to-moderate heritability (14–26%), resulting in a low efficiency of phenotypic selection, because of the major influence of environmental factors on phenotype. In addition, each year, approved stallions are re-evaluated during studbook inspections, and legwork is reassessed. This means that stallions that were inspected at the age of 3 years with an AA score may be withdrawn from stud service at later ages because of an inadequate CPL score. As a consequence, these stallions most likely have already been frequently used in breeding programs. Furthermore, mares that have not been inspected and scored are often used for breeding purposes.

François demonstrated that horses with high CPL scores can still genetically be less predisposed compared with horses with lower CPL scores, which is consistent with low heritability [10]. Selection could be improved by using estimated breeding values (EBV) [12]. EBVs are estimates of a horse’s genetic merit for a particular trait and an indication of how a horse’s progeny would perform. Using EBVs has two main advantages over phenotypic selection. Firstly, breeding values take into account differences in environment, nutrition, training, and other external factors that may affect performance. Thus, breeding values are corrected for environmental influences. Secondly, when estimating breeding values, not only the horse’s own performance but also the performance of all related animals is taken into account. Therefore, more information is available, allowing for better estimation of genetic potential [94]. The current phenotypical selection against CPL in the Belgian draft horse has not resulted in a negative trend (towards less CPL) in the average EBV of CPL in the approved breeding stallions [10]. Although the maximum levels of CPL scores seem to have decreased (but not the prevalence), so has the variation of the EBVs, which additionally compromises selection that is based on EBVs [10].

Other major concerns in the Belgian draft horse population are the high level of inbreeding and the low effective population size, resulting in low levels of genetic diversity. According to Janssens et al., the effective population size of the Belgian draft horse (based on data of the KMBT) is estimated to be 48 animals (2020), when calculated based on the increase in coancestry [95]. For domesticated breeds, the effective population size must be above the safe lower limit of 100 animals, while an effective population size of 50 animals is considered the absolute critical lower limit. Below these limits, inbreeding and coancestry rates will increase at excessive rates, posing a high risk of the breed’s extinction in the short term [96]. This limited genetic diversity puts the Belgian draft horse breed at serious risk and complicates the additional selection against a genetic disorder without further limiting genetic variation in the population.

## 8. Conclusions and Perspectives

Chronic progressive lymphedema in feathered draft horses is a disorder that, due to its high prevalence and clinical implications, has a significant impact on the survival of several breeds, including the Belgian draft horse. Clinical signs are severe, compromise the horse’s quality of life, affect animal welfare, and even cause a decreased life expectancy. The treatment and management of CPL focus on preventing and treating secondary infections, and aim to slow down the disease’s progression. Despite the severity of this condition, many uncertainties regarding its etiology, pathogenesis, and subsequent possible treatment still remain to date. Chronic inflammation could represent an important factor in the pathogenesis of CPL, in addition to progressive fibrosis, an aberrant elastin network, and lymphedema. The chronology of these factors currently remains one of the main points of discussion. Established scientific research on CPL is rather limited, despite the urgent need for answers. Several aspects of this condition require further exploration and the possibilities for future research are numerous.

## Figures and Tables

**Table 1:** CPL scoring system by Colman and De Keyser (2010). This scoring system was developed by Colman and De Keyser (2010) and is currently being used in practice for the assessment of Belgian draft horse stallions during studbook approvals. Adapted with permission from Colman, J., De Keyser, K. (2010). Het scoren van de beenkwaliteit bij het Belgisch trekpaard. Ledenblad K.M.B.T. 84, 40.

Grade	Severity	Region	Clinical Signs		
			Skin Lesions	Skinfolds/Nodules	Soft Tissue, Skin and Hairs
AA			- None	- None - Normal limb diameter (25–30 cm below carpus, 33–38 cm under the hock)	- No swelling - Hairs and skin are supple
A	Mild	Below fetlock	- Sometimes slight skin thickening and scaling - (Hock: hairs upright)	- 1 to 2 skinfolds in the pastern cavity - Normal limb diameter	- Slight, mild skin thickening, impressionable
B	Moderate	Up to fetlock	- Moderate skin thickening and scaling, sometimes wounds - Hock and fetlock: hairs upright with moderate lesions - (Exudate on the hock)	- >2 skinfolds in the pastern region (palmar/plantar or dorsal) - Nodules in the fetlock region - Normal limb diameter	- Moderately hard, diffuse swelling
C	Severe	Above fetlock	- Severe skin thickening and scaling, wounds - Hocks with wounds, exudate and hairs upright - Slight mechanical impairment	- Skinfolds palmar/plantar and/or dorsal - Nodules ascending - Increased limb diameter	- Hard, diffuse swelling - Hairs are rough and broken
D	Extreme	Above fetlock	- Very severe skin thickening, scaling, wounds, blood, exudate and bad odour - Hock with open wounds and hairs upright - Severe mechanical impairment	- Skinfolds and nodules surrounding the limb - Increased limb diameter	- Hard, diffuse swelling - Hairs are rough and broken

## Data Availability

Data sharing is not applicable to this article as no new data were created in this study.
